# Prevalence of Transmitted HIV Drug Resistance in Iran between 2010 and 2011

**DOI:** 10.1371/journal.pone.0061864

**Published:** 2013-04-23

**Authors:** Fatemeh Jahanbakhsh, Junko Hattori, Masakazu Matsuda, Shiro Ibe, Seyed-Hamid R. Monavari, Arash Memarnejadian, Mohammad R. Aghasadeghi, Ehsan Mostafavi, Minoo Mohraz, Hossain Jabbari, Kianoush Kamali, Hossein Keyvani, Kayhan Azadmanesh, Wataru Sugiura

**Affiliations:** 1 Department of Virology, Tehran University of Medical Science, Tehran, Iran; 2 Department of Virology, Pasteur Institute of Iran, Tehran, Iran; 3 Clinical Research Center, Nagoya Medical Center, Nagoya, Japan; 4 Department of Hepatitis and AIDS, Pasteur Institute of Iran, Tehran, Iran; 5 Department of Epidemiology, Pasteur Institute of Iran, Tehran, Iran; 6 Infectious Diseases Department, Iranian Research Center for HIV/AIDS (RCHA), Tehran, Iran; 7 Infectious Diseases Department, Iranian Research Center for HIV/ AIDS (RCHA), Digestive Diseases Research Institute, Tehran University of Medical Sciences, Tehran, Iran; 8 Vienna General Hospital, University, Department of Dermatology, Department of Clinical Immunology, Allergy and Infectious Diseases, Wien, Austria; 9 AIDS Office, Center for Communicable Diseases Control, Ministry of Health, Tehran, Iran; Centro de Biología Molecular Severo Ochoa (CSIC-UAM), Spain

## Abstract

**Objective:**

Drug-resistant (DR) HIV emerges during combined antiretroviral treatment (cART), creating concern about widespread transmission of DR-HIV as cART is expanded in resource-limited countries. The aim of this study was to determine the predominant HIV-1 subtypes and prevalence of transmitted DR mutations among antiretroviral-naïve patients in Iran.

**Design:**

To monitor transmission of DR HIV, a threshold surveillance based on the world health organization (WHO) guidelines was implemented in Iran.

**Methods:**

For this HIVDR threshold surveillance study, blood samples were collected from 50 antiretroviral-naïve HIV-1-infected patients. Antiretroviral-resistant mutations were determined by sequencing HIV-1 protease, reverse transcriptase and integrase regions. The HIV-1 subtype was determined by sequencing the p17 and C2-V5 regions of the *gag* and *env* genes, respectively.

**Results:**

Phylogenetic analyses of the sequenced regions revealed that 45 (95.7%) of 47 samples that were successfully obtained were CRF35_AD. The remaining two cases were subtype B (2.1%) and CRF01_AE (2.1%). Consistent results were obtained also from Env and Gag sequences. Regarding prevalence of transmitted DR viruses, two cases were found to harbor reverse transcriptase-inhibitor-resistant mutations (4.3%). In addition, although not in the WHO list for surveillance of transmitted mutations, 13 minor protease-inhibitor-resistant mutations listed in the International AIDS Society-USA panel of drug resistance mutations were found. No DR mutations were detected in the integrase region.

**Conclusions:**

Our study clarified that CRF35_AD is the major subtype among HIV-1-infected patients in Iran. According to the WHO categorization method of HIVDR threshold survey, the prevalence of transmitted drug resistant HIV in Iran was estimated as moderate (5–15%).

## Introduction

Selection and acquisition of HIV-1 drug resistance is inevitably associated with combined antiretroviral treatment (cART) of HIV-1-infected patients. Thus, the emergence of drug-resistant (DR) HIV is a major concern as a potential consequence of scaling up cART [1]. Indeed, expansion of cART might be jeopardized by widespread transmission of DR-HIV [2], particularly in countries where antiretroviral options are limited.

The first HIV-infected case in Iran was a hemophilic patient identified in 1986, and the first cases of HIV transmission through drug injections were reported in 1989 [3]. As of March 2012, 24290 HIV-1-infected individuals have been reported in Iran [4]. The transmission routes were attributed to injected drugs (69.6%), sexual contact (10.5%), transfusion of blood products (1.0%) and mother-to-child transmission (0.1%) [4]. Iran is experiencing a concentrated HIV-1 epidemic among injecting drug users (IDUs) [5], [6].

In Iran, cART was introduced in 1997 when the antiretrovirals zidovudine, lamivudine, and indinavir became available as a part of the country’s healthcare system, making it possible to scale up cART in Tehran and other cities such as Kermanshah and Shiraz [7]. Subsequently, indinavir was replaced with nelfinavir from the list of Iranian generic drugs. Later, stavudine, nevirapine, and didanosine in 2005, efavirenz, lopinavir/ritonavir, tenofovir, and abacavir in 2006, atazanavir in 2011 became available for HIV/AIDS patients [3], [7]. cART is in line with Iran’s guidelines on clinical care for HIV/AIDS patients, which state the 14 possible three-drug combinations from the antiretrovirals mentioned above [3]. cART is supplied to HIV/AIDS patients for free at counseling and behavioral centers (triangular clinics) [7].

In addition to receiving cART, all HIV/AIDS patients in Iran are monitored for CD4^+^ T cell counts periodically by the government at no cost for patients. On the other hand, the Iranian guidelines do not include measurement of viral load. Similarly, drug-resistance monitoring is available on a limited basis, and it is not tested on every Iranian patient for drug resistance. Therefore, to address concerns about the emergence and transmission of DR-HIV in Iran, we conducted a threshold survey among drug-naïve individuals by following recommendations of the World Health Organization (WHO) drug-resistance network. In this study, we report the estimated prevalence of DR-HIV transmission in Iran.

## Methods

### Eligibility Criteria

Samples were collected according to the HIV Drug Resistance Threshold Survey (HIVDR-TS) recommended by the WHO for the surveillance of DR-HIV in resource-limited countries [8]. Individuals were recruited from all counseling and behavioral centers in Tehran from January 2010 through February 2011 if they met these eligibility criteria: under age 25 years at HIV diagnosis and no previous pregnancy for females [8]. However, after these 9 months, only 15 newly diagnosed cases were recruited. Thus, after consultation with WHO experts, to reduce sample collection period, the inclusion criteria were expanded to less than 30 years of age, CD4^+^ T cell counts >500/µl, without previous pregnancies for females and no previous exposure to antiretroviral drugs. In addition to Tehran, samples were collected from two other areas in Iran, Kermanshah in the west and Shiraz in the south of Iran, where antiretroviral therapy was started at the same time as Tehran.

### Sample

Newly diagnosed, antiretroviral-naïve HIV-1 patients (n = 50) were enrolled in this study. They visited counseling and behavioral centers in Tehran (n = 30), Kermanshah (n = 10), or Shiraz (n = 10) between January 2010 and February 2011. After patients signed informed consent, 10 ml of their peripheral blood was collected into EDTA-containing vacutainer tubes. Plasma samples were obtained by centrifugation and aliquots were stored at -70°C until use. If possible, each patient was asked to complete a questionnaire regarding patient’s basic information, including age, sex, risk behavior, marital status, and status of hepatitis B virus (HBV) or hepatitis C virus (HCV) co-infection.

### Ethics Statement

This study was approved by the Ethics Committees in Medical Sciences Research at the Tehran University of Medical Sciences and Ministry of Health and Medical Education.

### Determination of Drug-Resistance Mutations by Genotyping

We used the drug resistance genotypic testing protocol described previously by Sugiura, et al. with some modification [9]. Briefly, viral RNA was extracted from 140 µl of plasma by the QIAamp Viral RNA Mini Kit (Qiagen, Tokyo, Japan) according to the manufacturer’s instructions. Nucleotide sequences of whole HIV-1 protease (PR, HXB2 position 2253–2549), the N-terminal portion of reverse transcriptase (RT, 2550–3269), and full-length integrase (INT, 4230–5093) were amplified using region-specific primer pairs by reverse transcription (RT)-polymerase chain reaction (PCR) using SuperScript III one-step RT-PCR system with platinum Taq high-fidelity kit (Life Technologies Corp., Tokyo, Japan), and amplified further in nested PCR using LA Taq (Takara Bio Inc., Shiga, Japan). After gel electrophoresis, the amplified PCR products were purified by QIAquick Gel Extraction Kit (Qiagen, Tokyo, Japan). Sequencing reaction was performed using the BigDye terminator cycle sequencing kit v3.1 (Applied Biosystems, Tokyo, Japan). Nucleotide sequences were determined on the ABI PRISM 3130 Genetic Analyzer (Applied Biosystems). The sequenced data were aligned against HXB2 and amino acid mutations were detected by SeqScape 2.5 software (Applied Biosystems). Presence of transmitted HIV-1 drug-resistance mutations was determined using the WHO mutation list [10].

### HIV-1 Subtype Determination

To determine the subtype of each sample, the nucleotide sequences including p17 of the *gag* gene (Gag, HXB2 position 708–1230) and the C2-V5 region of the *env* gene (Env, 6934–7651) were determined using the same protocol as for the drug-resistant genotypic test described above. The nucleotide sequences obtained for Gag and Env as well as PR, RT, and INT were aligned by ClustalW, and phylogenetic trees were constructed using neighbor-joining analysis with 1000 bootstraps as implemented in MEGA 5 [11].

### Nucleotide Sequence Accession Numbers

The nucleotide sequences obtained in this study were deposited in the DNA Data Bank of Japan, and are available under the following accession numbers: AB716095–AB716141 for PR and RT, AB716142–AB716188 for INT, AB716189–AB716232 for Gag, and AB716233–AB716276 for Env. To be noted, one of the *env* sequences contained ambiguous regions. Thus, it was submitted in two parts: AB716271 and AB716272.

## Results

### The Major HIV-1 Transmission Route in Iranian Cases is Injecting Drug Use

Background information on the 50 newly diagnosed HIV-1-infected cases is summarized in Table 1. More than a half of study cases were male (64.0%) and in the 25–30 year-old age group (56.9%). Their average age was 26.0 years. Among 35 participants who completed the questionnaire, 51.4% were infected by drug injections. Together with 9 individuals who acquired HIV through heterosexual contact with IDU partners, IDU-related transmission accounts for 77.1%. Co-infection with hepatitis C virus and hepatitis B virus was identified among 46.7% and 7.1% of 30 and 28 tested participants, respectively.

**Table 1:** Baseline characteristics of newly diagnosed HIV-1 infected patients in Iran.

Characteristic	*n* (%)	
Men^1^	32	(64.0)
Age (years) 1		
<20	2	(4.0)
20–24	13	(26.0)
25–30	35	(70.0)
Residence^1^		
Tehran	30	(60.0)
Kermanshah	10	(20.0)
Shiraz	10	(20.0)
Marital status^2^		
Single	20	(57.1)
Married	11	(31.4)
Divorced	3	(8.6)
Widowed	1	(2.9)
Transmission route^2^		
Injecting drugs	18	(51.4)
Heterosexual contact	6	(17.1)
From injecting drug-using husband	8	(22.9)
From injecting drug-using wife	1	(2.9)
Unknown	2	(5.7)
Coinfection^3^		
Hepatitis C virus	14	(46.7)
Hepatitis B virus	2	(7.1)

1 Information on gender, age, and city of residence were obtained from all 50 cases who completed the questionnaire.

2 Information on marital status and transmission route were obtained from 35 cases who completed questionnaires.

3 Infection with hepatitis C virus and hepatitis B virus were tested in 30 and 28 cases, respectively.

### The Predominant HIV-1 Subtype is CRF35_AD

**Figure 1:**
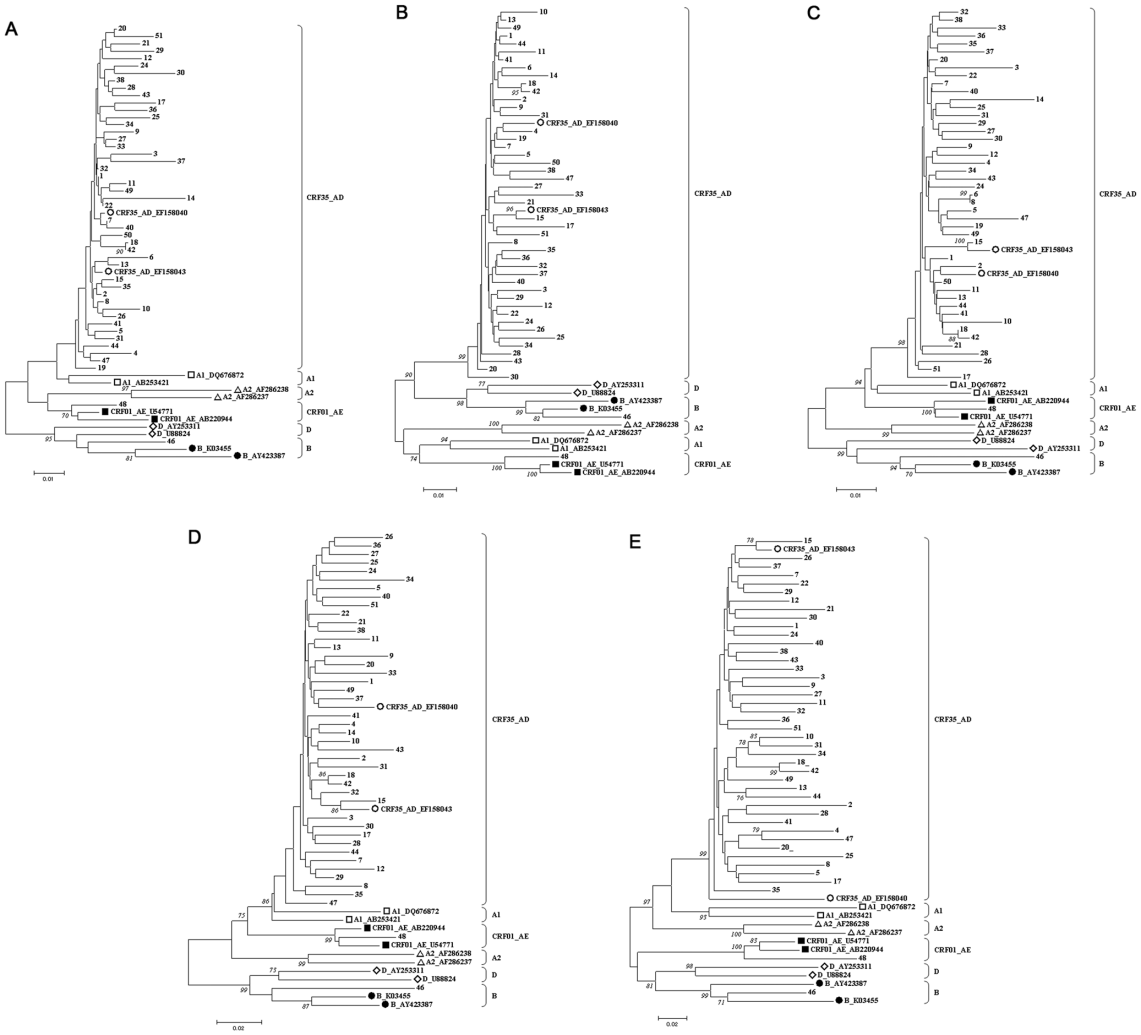
Phylogenetic trees of sequenced Iranian samples. (A) protease, (B) reverse transcriptase, (C) integrase, (D) gag, and (E) env. Trees were constructed using neighbour joining method with 1000 replicates. Two sequences each of 6 HIV-1 subtypes retrieved from the GenBank are included as references. Open circles (○) indicate the reference sequence of CRF35_AD, open squares (□) indicate subtype A1, open triangles (▵) indicate A2, open diamonds (◊) indicate subtype D, closed circles (•) indicate subtype B, and closed squares (▪) indicate CRF01_AE. Bootstrap values over 70% are shown.

Among 50 collected specimens, 47 were successfully sequenced in the PR, RT and INT regions (94.0%). Phylogenetic analyses of these PR, RT and INT sequences clarified that 45 of 47 samples were CRF35_AD (95.7%), one was subtype B (2.1%), and one was CRF01_AE (2.1%) (Figures 1A–C). Amplification and nucleotide sequences of Gag and Env were successfully obtained from 44 (88.0%) and 43 (86.0%) specimens, respectively. Consistent results were obtained from phylogenetic analyses of Gag and Env sequences, and no discrepant case was found (Figures 1D, E).

### Prevalence of Transmitted Drug-resistant Mutations of Our Study Population is Moderate

Surveillance drug-resistant mutations (SDRMs) associated with transmitted HIV-1 drug resistance [10] were detected in the RT gene of two specimens. Both SDRMs were related to nucleotide reverse transcriptase inhibitor (NRTI) resistance mutations: T215D found in a patient from Kermanshah and K219Q in a patient from Tehran (Table 2).

**Table 2:** Prevalence of drug resistance-associated mutations among 47 sequenced samples.

SDRMs	*n*	Frequency (%)
NRTI-resistant mutations		
T215D	1	2.1
K219Q	1	2.1
**IAS-USA**		
PI-resistant mutations		
L10I/V	5	10.6
V11I	2	4.3
G16E	3	6.4
K20R	46	97.9
L33V	1	2.1
M36I	47	100.0
D60E	2	4.3
I62V	5	10.6
L63P	1	2.1
I64L/V	3	6.4
H69K	46	97.9
L89M	46	97.9
I93L	1	2.1

IAS-USA, the international Antiviral society-USA; NRTI, nucleoside reverse transcriptase inhibitor; PI, protease inhibitor; SDRMs, surveillance drug-resistant mutations.

Other than the SDRMs in the WHO list [10], 13 minor mutations in the PR gene listed in the IAS-USA panel [12] were detected (Table 2). M36I was found in all cases (100%), H69K and L89M were found in all except one subtype B sample (97.9%), and K20R was found in 46 samples (97.9%), and they are considered as polymorphic to non-B HIV. In addition, L10V, V11I, G16E, L33V, D60E, I62V, L63P, I64L and I93L were detected, but were less common. Within the INT gene, we found no drug resistance-associated mutation listed in among the IAS-USA panel [12].

## Discussion

Here we report the circulating HIV-1 subtype and prevalence of DR-HIV among 50 newly diagnosed HIV-1-infected cases in three different cities of Iran. Among 47 sequenced samples, 45 samples (95.7%) were determined as CRF35_AD, one sample (2.1%) as subtype B and another (2.1%) as CRF01_AE. Our results are consistent with two recent reports on the predominance of CRF35_AD in Iran [13], [14]. Before 2007, subtype A1 had been reported as the predominant HIV-1 subtype in Iran [15]–[17]. However, when we obtained these HIV-1 Env and Gag sequences registered as subtype A1 before 2007 and conducted phylogenetic analysis, we found that they make an evident cluster with CRF35_AD (data not shown). This result suggests that CRF35_AD has been circulating in Iran even before 2007. Taken together, these results indicate that the predominant HIV-1 in Iran is CRF35_AD.

Based on the WHO surveillance list for transmitted resistance [10], only two SDRMs, K219Q and T215D, were found in this study. K219Q predict potential low level resistance to zidovudine and stavudine, and T215D is a revertant mutation associated with an increased risk of virologic failure to TAMs. The prevalence rate calculated from our sample was 4.3% (2/47). Applying the WHO HIVDR-TS formula [18], the prevalence of SDRMs in the RT gene in Iran can be classified as moderate (5–15%). This finding is not surprising considering that NRTIs have been available since 1997 and are widely distributed in Iran as main part of cART. Mutations conferring resistance to reverse transcriptase inhibitors (NRTI and NNRTI) are most common form of transmitted drug resistance detected worldwide, while protease inhibitor resistance is generally less frequent. Two TDR mutations observed in this study were associated with the NRTI drug class. The transmission of NRTI resistance is worrisome since first-line antiretroviral therapy In Iran is based on NRTI+NNRTI combination since 2008. The low prevalence of transmitted drug resistance to PIs was expected giving that the access to these drugs has been limited in Iran [7]. In fact, within the PR gene, we found no mutations associated with SDRMs in our studied cases. However, these specimens had 13 minor drug-resistance mutations based on the IAS HIV-DR list of mutations [12]. Four mutations, K20R, M36I, H69K, and L89M were found in high prevalence (97.9%, 100%, 97.9%, and 97.9%, respectively), consistent with previous reports [13], and can be considered polymorphisms for the predominant subtype CRF35_AD in Iran. Thus, the prevalence of SDRMs in the PR gene was classified as low (<5%). It is likely that transmission of HIV-DR in PR is still uncommon.

A recent pilot surveillance study of HIV drug-resistance transmission in Iran found SDRMs in the RT gene among 39 sequenced specimens; D67D/G was found in a specimen from Esfahan, and V75A/V was found in a 5-year-old female from Tehran both of which confer resistance to NRTIs [13]. Although the sample for this pilot threshold survey was too small to estimate the prevalence of SDRMs, finding two SDRMs in the RT gene is remarkable. Besides CRF35_AD, a subtype G and an A HIV-1 were found in pilot study, whereas in this study, one subtype B and one CRF01_AE cases were found, which suggest that these minor strain are not spreading rapidly.

Although increasing number of individuals is receiving ART in Iran, the coverage rate was still low amoung those in need of therapy in December 2011 [7]. It is even lower among IDU because physicians are concerned that injecting drugs is considered as a risk factor for poor adherence which results in development of DR HIV, and they hesitate to prescribe antiretrovirals [19], [20]. Considering low cART coverage among IDU, who comprised the majority of our major study sample, the moderate (5–15%) prevalence of SDRMs estimated by applying the WHO HIVDR-TS leads us to suspect that frequent and efficient DR transmission is taking place within the IDU population. As more eligible patients actually start to receive cART, it is assumed that the prevalence of DR HIV may increase. Thus, it is important to review monitoring data on antiretroviral therapy programs for the relevant geographical area and investigate potential problems with regard to several factors: continuous access to services, drug supply, drug quality, prescribing practice toxicity, adverse events, drug sharing, and treatment failures.

In conclusion, according to the WHO categorization method of HIV-drug resistance threshold survey, the prevalence of transmitted drug resistant HIV in Iran was estimated as moderate. This indicates that transmitted drug resistance surveillance should be repeated in 1-year period, according to the WHO guidelines.
